# The West Midland Region Meeting at Birmingham

**Published:** 1920-09-25

**Authors:** 


### THE BRITISH HOSPITALS ASSOCIATION.

### The West Midland Region Meeting at Birmingham.

A meeting of the West Midland region of the British Hospitals Association, which comprises the counties of Hereford, Salop, Stafford, Warwick, and Worcester, was held at the General Hospital, Birmingham, on the 16th inst. The chair was taken by the Hon. Sir Arthur Stanley, G.B.E., President of the Association.

The President, in opening the meeting, said that, in view of threatened legislation and other evils, it was felt necessary to strengthen the Association. Whilst there was a good association of Poor-law Unions, there was none, except the British Hospitals Association, which could speak in the name of the voluntary hospitals, and the Ministry of Health welcomed such a body, because they could readily refer to it to ascertain the general opinion of the voluntary hospitals of the country. Instead of trying to concentrate everything in London, the country had been divided up into regions, and meetings were now being held with the view of discussions, by means of which it was hoped to ascertain the general feeling of the country.

### Dr. Addison and the Voluntary System.

Many people were of opinion that, because Dr. Addison had got in his new Bill a clause enabling hospitals to be put on the rates it was therefore in the mind of the Minister to take this step. Having heard Dr. Addison speak on this subject several times, he was able to say that this was not the Minister's view. The clause was simply permissive, and Dr. Addison was most anxious to keep the voluntary system. Nobody would be more pleased than Dr. Addison himself if any efforts put forth were successful in preventing this additional burden being put on the Treasury or on the rates.

Sir Napier Burnett said finance was at the bottom of the whole question, and if it could be shown that the voluntary hospitals could get enough money to carry on nobody need worry about interference from the Government. Returns made by sixty hospitals in the West Midland region showed an ordinary income for the five years 1915-19 of £1,696,758 and an ordinary expenditure of £1,874,181, leaving a deficit of £177,423 on the five years' working. But, in addition to the ordinary income, these hospitals received during the same period 209,879 as free or unearmarked legacies, so that this group received during the five years £32,456 more than was required to meet their ordinary expenditure. Excluding these legacies, of course, practically all the hospitals showed considerable deficits, and he estimated that, taking into account all the hospitals of the country, something like a million pounds was required to put them back into the position they occupied in 1914. In 1919, for reasons well known, while expenditure continued to advance, income remained stationary, or actually receded. What was worrying the hospitals at the moment was where the income was to come from to meet the present expenditure.

### Present Appeal Methods Wasteful.

Sir Napier Burnett said he could see no relief from this anxiety while so many hospitals relied on their present methods of raising money—namely, by circulars and spasmodic appeals. These methods were losing their power, and the public were becoming wearied by the multiplicity of such appeals. What was wanted was to supplement these by a regular stream of weekly collections from the employees of the factories, workshops, and business houses, and, in like manner, guaranteed amounts from the employers. Workpeople generally were willing to adopt this form of voluntary levy, but the hospitals must create the machinery for its collection. Hospitals in the West Midland group received, in the shape of workpeople's contributions, sums ranging from £1 per available bed up to £51 per available bed. He gave these examples not by way of criticism, but to indicate the need there was for further work in this direction. Points requiring consideration were that a hospital patient had a value to his wife and family, to his employer, to insurance companies, to the State, and to the municipality in which he lived. There was need for educational propaganda to inform the public of the work being done by the hospitals.

### Payments by Patients.

With regard to paying beds, he had been surprised during his recent survey of the hospitals to find that quite a large number in the provinces had made arrangements for paying patients, but many of the larger hospitals had still made no provision by which people with fixed limited incomes could be admitted for treatment at modified charges. These people could not afford the expense of a private nursing home, but they desired to pay their way—a British instinct which might be encouraged rather than repressed or ignored. There was money waiting to be picked up from patients, either through the almoner's system or by a fixed levy per head per week towards maintenance, or by paying beds. Passing next to the patient's value to the employer, it could be said that curative medicine, as a legitimate tax upon industry, had not been forcibly brought to the notice of the commercial community. Comparatively few business firms appreciated the great volume of work of an economic value which the voluntary hospitals were doing. One secretary wrote to the effect that all employers who overstepped the demands on the hospital to any appreciable extent were sent an account and asked outright if they were aware of the use they were making of other people's subscriptions. This was an example that should be widely copied. He feared there was a degree of inertia possessing some hospital administrators which threatened to lower the flag of the voluntary principle.

### Insurance Companies and Subscriptions.

Next, the hospitals benefited the general insurance companies, accident offices in a direct manner, and life offices indirectly. If hospitals were to close their doors there would in all probability be a considerable increase in the amount to be paid out on compensation for sickness and accident, and even on life policies themselves. Yet these wealthy insurance corporations rendered but a meagre financial return for all the valuable work done for them. Of a quarter of a million ordinary income in 1918, the insurance companies subscribed less than £300. An appeal sent out by one secretary in 1919 to fifty-three insurance companies brought forth but one answer, accompanied by a donation of £20. The secretary added quaintly, "Fifty-three were cured, but only one returned to give thanks." (Laughter.) Whilst the hospitals exercised a distinct advantage to the community in healing the sick and training doctors and nurses, yet municipalities made no adequate return in the shape of remuneration.

(*Continued on p.* vi.)

Whilst they were supposed to treat all street accidents, the hospitals got no financial return, but were supposed to buy commodities and pay their bills as best they could. The fact was there were burdens being put on the back of the voluntary principle that it was never intended to carry, and the hospitals should now arouse themselves and see that these additional burdens were removed.

The View of the General Public.

While the general public would always be ready to subscribe to an institution that treated those who could not pay for themselves, many were hesitating to subscribe to hospitals treating so many patients who could afford to pay or be paid for. As to the value of the hospital patient to the State, he suggested that whatever tended to increase the non-effective group of workers at the expense of the effectives was a matter of serious national concern, and it was surely part of the responsibility of the State to see that the necessary hospital accommodation was provided for those injured in producing and building up the wealth of the nation. He had no hesitation in asserting that some form of State subsidy was required in return for the work which the hospitals were doing for the welfare of the State; but it should bear no direct relationship to maintenance, for as a capitation grant increased so would voluntary support recede, until in the course of a few years the hospitals would be financed entirely by the State. The subsidy should be limited to provide the requisite hospital accommodation, a certain amount of equipment, payment towards the cost of training nurses, and the encouragement of pathological and bacteriological research. Municipally-controlled hospitals had proved to be more costly than voluntary institutions. He suggested that in order to bridge over present difficulties some of the Poor-Law hospitals should be loaned or handed over for use as general hospitals.

Mr. C. H. Saunders (Birmingham) was appointed Chairman of the Region, and Mr. T. B. Adams (Wolverhampton), Vice-Chairman.

